# Infant regulatory problems and the quality of dyadic emotional connection—a proof-of-concept study in a multilingual sample

**DOI:** 10.3389/frcha.2023.1304235

**Published:** 2024-01-05

**Authors:** Julia Jaekel, Anne-Kathrin Dathe, Maire Brasseler, Johanna Bialas, Elina Jokiranta-Olkoniemi, Margarete Reimann, Robert J. Ludwig, Amie A. Hane, Martha G. Welch, Britta M. Huening

**Affiliations:** ^1^ Unit of Psychology, Faculty of Education and Psychology, University of Oulu, Oulu, Finland; ^2^ Department of Paediatrics I, Neonatology, Paediatric Intensive Care, Paediatric Neurology, University Hospital Essen, University of Duisburg-Essen, Essen, Germany; ^3^ Department of Health and Nursing, Occupational Therapy, Ernst-Abbe-University of Applied Sciences, Jena, Germany; ^4^ Center of Translational Neuro- and Behavioural Sciences, C-TNBS, Faculty of Medicine, University of Duisburg-Essen, Essen, Germany; ^5^ Department of Pediatrics, Columbia University Irving Medical Center, New York, NY, United States; ^6^ Department of Psychology, Williams College, Williamstown, MA, United States; ^7^ Department of Psychiatry, Columbia University Irving Medical Center, New York, NY, United States; ^8^ Department of Anatomy and Cell Biology, Columbia University Irving Medical Center, New York, NY, United States

**Keywords:** regulatory problems, dyadic interactions, emotional connection, behaviour observation, linguistic heterogeneity

## Abstract

**Background and aims:**

Close autonomic emotional connections with others help infants reach and maintain homoeostasis. In recent years, infant regulatory problems (RPs, i.e., crying, sleeping, and feeding or eating problems) have surged. This study has two aims: (1) Provide proof-of-concept that dyadic autonomic emotional connection between infants and parents can be reliably assessed with a brief screening, irrespective of language and culture. (2) Assess in a heterogeneous pilot sample whether the persistence of RPs during infancy is negatively associated with the quality of dyadic autonomic emotional connection.

**Methods:**

30 children aged 3–68 months (47% female) and their parents (83% mothers) were assessed during regular neonatal follow-up visits in Germany. Seven (23%) dyads were immigrants whose primary language was not German. At each assessment, paediatricians asked parents about infant's crying, sleeping, and feeding or eating problems. Dyadic interactions were rated by a multilingual team with the standardised universal Welch Emotional Connection Screen (uWECS) on four dimensions (attraction, vocalisation, facial communication, sensitivity/reciprocity).

**Results:**

Aim 1: An international team of raters was trained remotely to rate the uWECS. Reliability of *α *>* *.90 with standard raters was achieved irrespective of language mismatches (i.e., all raters scored several videos with languages they did not understand). Intra-class correlation coefficients (*ICCs*) among five main raters for the four uWECS dimensions ranged from .98–.99. Aim 2: Infants (*n *= 15 assessed longitudinally) had mean RP scores of 1.20 (SD* *= 1.26). Dyads had mean uWECS scores of 7.06 (SD = 2.09). Linear regression analysis showed that more persistent RPs in infancy were associated with lower uWECS scores [*β *=* *-.53, *95% CI *= (-1.47, -.18), *p *= .017], after controlling for child sex and gestational age.

**Conclusion:**

This study provides proof-of-concept that the quality of mutual autonomic emotional connection among socio-culturally and linguistically heterogeneous samples can be reliably assessed with the uWECS, a brief screening that can be easily implemented in clinical practice. Pilot data suggests that persistent RPs during infancy are negatively associated with the quality of dyadic autonomic emotional connection. Replication of these findings in larger samples is warranted. Future studies need to address how to facilitate successful emotion regulation for today's children and future generations.

## Introduction

Societies and human life across the world are rapidly changing due to factors such as global warming and increased migration. Across cultures, our species' evolution and survival universally depend on the formation of close relationships with others (1, 2). Ludwig and Welch (3) have proposed that social interaction between mother and infant is a dynamic process that already starts *in utero* and follows a conditional autonomic socioemotional reflex (ASR) and emotional connection. The ASR is dynamically shaped by reciprocal learning and co-regulatory change via various biological cycles, such as sleep-wake, signalling, feeding, and breathing (3). These co-regulatory or calming cycles of emotional connection are intended to facilitate homoeostasis, and their quality has profound impact on infant development that can last life-long (3). Here, mother-infant autonomic emotional connection is qualitatively distinct from the widely used more colloquial or “psychological” emotional connection construct (4). However, rates of both types of emotional connection have been low in recent years, while infants and parents have been under severe mental stress (5–7). Simultaneously, infant regulatory problems (RPs, i.e., crying, sleeping, and feeding or eating problems) have surged in recent years (8, 9), with many parents seeking support from health professionals. Research suggests a continuous feedback loop, with infant RPs increasing parenting stress (10), while stressed parents may respond with poorer parenting quality (11, 12), which may then further exacerbate poor regulatory behaviour in their children (13). In light of these challenges for today's families, we lack information about the association between infant regulatory problems and the quality of emotional connection with their parents.

Autonomic emotional connection is dependent upon vocal signalling, feeding, and sleeping, which are fundamental for infant survival and healthy development (14). Persistent difficulties with self-regulating these behaviours may result in RPs such as waking up many times and not settling back to sleep at night or neophobia to food (15, 16). Most early RPs are transient, but a combination of multiple problems or persistent RPs are associated with high risk for behaviour regulation difficulties (17–22) and emotional disorders later in life (23). The definition of infant RPs includes excessive crying after 3 months of age, as well as feeding and/or sleeping problems after 6 months of age (24). Approximately 20% of infants experience some of these RPs (18, 25), while previous studies have found that 2%–9% may have persistent RPs across more than one assessment point (15, 24, 26).

In general, self-regulation is a multifaceted construct including complex bodily and behavioural-cognitive functions. Of these, emotion and behaviour regulation are particularly relevant for children's school readiness, wellbeing, and life-course success (27, 28). Consequently, researchers, practitioners, and families alike are highly interested in environmental factors such as parenting that can support children's growing self-regulatory skills. For instance, maternal sensitivity, defined as adaptive, prompt, and responsive parenting has been found to foster dyadic co-regulation of behaviour (29, 30). However, social interactions are bidirectional in nature, and characterised by constant feedback loops. Accordingly, early social behaviour and caregiving should not be seen as unidirectional but instead assessments should capitalise on the bidirectionality and mutuality in the exchange of behaviours (1, 31). Here, two key features are synchrony and autonomic emotional connection, i.e., the dynamic and reciprocal adaptation of behaviours, co-regulation, and shared affect between two interactive partners (32). High synchrony is associated with healthy development across multiple dimensions (33), while early-life risks such as maternal chronic stress or preterm birth are negatively associated with dyadic synchrony (29, 34–37).

Synchrony and mutual autonomic emotional connection are not culturally-specific constructs, they are universal and form fundamental building blocks of human behaviour. In light of growing cultural and linguistic diversity worldwide due to increased migration (38, 39), the availability and implementation of equitable assessment tools that use a universally accessible language has become paramount. Minimal and easy language approaches provide solutions for overcoming some of the complexity, ambiguity, and cultural variability of emotion words (40), so that assessment tools can be valid and reliable for use in a wide range of countries and professional settings.

The original Welch Emotional Connection Screen (*WECS*) (31, 40) was created to specifically assess the parent-child autonomic emotional connection construct. In this study, we use a version of the WECS that was made universally accessible using a minimal language version with Clear Explicit Translatable Language (31, 40), now referred to as the universal *uWECS*.

The current study has two aims:

(1) Provide proof-of-concept that dyadic autonomic emotional connection between infants and parents can be reliably assessed with the brief uWECS screening, irrespective of raters' and participants' languages and cultures.
(2) Assess in a pilot sample whether the persistence of RPs during infancy is negatively associated with the quality of dyadic autonomic emotional connection. We hypothesized that infants with more persistent RPs would have lower quality of dyadic emotional connection with their mothers or fathers.

## Materials and methods

Data were collected as part of a retrospective single centre cohort study. The study protocol was approved by the ethics committee of the University Duisburg-Essen (23-11268-BO). Children aged 3–68 months and their parents were assessed as part of regular neonatal follow-up visits at a children's hospital in a large metropolitan region in Western Germany. Assessments with infants aged 24 months and younger were corrected for prematurity, while assessments with children older than 24 months were carried out according to chronological age.

### Regulatory problems

As part of neurodevelopmental examinations during regular neonatal follow-up visits at 3, 6, and 12 months of age, paediatricians asked parents about their infant's crying, sleeping, and feeding or eating behaviour. Definitions of crying, sleeping, and feeding or eating problems were derived from previous studies (24, 41). Specifically, paediatricians entered free text of the specific problems as described by parents at each visit, including intensity, frequency, and situational context into the database. Text chunks were extracted and jointly coded by a multiprofessional team (BM, JJ) according to the predefined protocol. Specific problems ranged from excessive, prolonged (>3 h daily) and intensive crying without apparent reason resulting in bluish lips and hands at 3 months, to problems with sleeping through the night, frequent waking and difficulties falling back to sleep at 12 months, or very picky eating or refusal of solid food, for example. The occurrence of clinical problems in one or more areas at an assessment was coded as 1 (vs. 0 = no problem). These were then summed into a variable that indicated the persistence (0 = never to 3 = RPs at all assessment points throughout infancy).

### Dyadic interactions

Video recording was performed to assess the quality of emotional connection between child and parent either before medical and standardised testing during follow-up or at the end of the appointment, depending on the regulatory state of the child (i.e., awake and not hungry at the start of the interaction recording). Parents were instructed to hold their child on their lap so they could be in good eye contact with each other. They were asked to interact with their child as they normally would for 3–5 min in their mother tongue (L1) without using any objects, toys, or food. The video camera was positioned on a tripod so that the child's face could be seen well in profile, as could the parent's face while the dyad was sitting on a chair. Sounds and speech were recorded via an integrated microphone. The dyad was left alone in the room for the observation period, but a staff member was nearby in visual and/auditory contact.

### uWECS

Video-recorded dyadic interactions between children and parents were rated by a multilingual international team with the Welch Emotional Connection Screen in Clear Explicit Translatable Language (WECS-CETL) (31, 40, 42), now referred to as the Universal WECS (uWECS) (see Hane et al., this special edition). The team of trained raters consisted of multiple professions, including neonatologists, psychologists, an occupational therapist, and a paediatric nurse. The WECS is a short interactive task and coding system, it has very good concurrent and construct validity. Higher emotional connection is correlated with healthier infant autonomic and behavioural stress responding (31). The WECS has been validated for infancy and into preschool age (31, 43, 44). The WECS includes four continuous dimensions (*attraction*, *vocalisation*, *facial communication*, and *sensitivity/reciprocity*) that were translated from the CETL words of the uWECS into the different languages represented among the coders according to a structured group process of translation within the training context as needed. Like the WECS, the uWECS includes dimensional scores and a binary rating of the dyad's emotional connection (yes/no; see Supplementary Appendix 1 for a parallel presentation of the full English and German uWECS positive and negative dimensional coding descriptions).

Each dimension was coded on a scale from 1 (lowest) to 3 (highest) according to 0.25 intervals (see Supplementary Appendix 2). *Attraction* was coded according to bidirectional gaze, physical proximity to each other, and mutual touch. Higher scores were given for gazing at, leaning into, and touching or reaching for the other with the goal of maintaining or establishing a connection. Gaze aversion or physical avoidance received low scores. *Vocalisation* was rated according to mutual warmth in vocal tone, amount of vocal stimulation from the mother, vocal or behavioural responsiveness from the child, and overall reciprocity of utterances. A high score was given to dyadic partners whose vocal behaviour was directed to the other to establish or maintain a connection. A low score was given for silence, lack of reciprocity, a negative/harsh tone of voice, or prolonged infant fussing or crying. *Facial communication* was coded according to mutually positive emotions expressed with the face such as smiling, joy, and empathy. Low scores were given for flat or negative facial affect, or when expressed emotions were not reciprocally matching each other. *Sensitivity/Reciprocity* was determined based on the dyad's social sensitivity to each other's expressed emotions or anticipated/identified needs. Harmonious and synchronous interactions received high scores.

Reliability analyses (Aim 1) were carried out on the individually scored dimensions. Here, by design, this proof-of-concept study included language mismatches such that each rater scored several videos of dyadic interactions in languages they did not understand. For Aim 2, the scores on each dimension were averaged across all raters and then summed into one continuous uWECS score (range 4–12). For the final binary rating, in line with previous studies (31), dyads with a continuous uWECS score ≥9 were considered emotionally connected.

### Biological and medical characteristics

Information on infant sex, perinatal medical risks, and age at assessment was retrieved from medical records.

### Parent questionnaires

Mothers and fathers answered a set of demographic and psychosocial background questions, including information on their level of education according to the International Standard Classification of Education (ISCED) (45), mother tongue (L1), and country of birth.

## Results

Table 1 shows that the 30 children in the sample had comparatively high perinatal risks and were of diverse backgrounds. Seven (23%) dyads had an immigrant background and spoke a first language other than German during the uWECS assessment. The subsample assessed longitudinally throughout infancy (*n *= 15) was characterised by equally high diversity.

**Table 1:** Descriptive sample characteristics.

	Total sample (*N* = 30)	Longitudinal subsample (*n* = 15)
Child sex [female, *n* (%)]	14 (47%)	10 (67%)
Gestational age [weeks, *M* (SD)]	30.80 (4.18)	29.60 (3.96)
Birth weight [grams, *M* (SD)]	1,660 (853)	1,500 (837)
Multiples [yes, *n* (%)]	11 (37%)	5 (33%)
Perinatal medical risks		
Intraventricular haemorrhage [IVH, grade 1–2, *n* (%)]	6 (20%)	3 (20%)
Periventricular leukomalacia [PVL, *n* (%)]	0	0
Necrotizing enterocolitis [NEC, *n* (%)]	1 (3%)	1 (7%)
Focal intestinal perforation [FIP, *n* (%)]	2 (7%)	0
Respiratory distress syndrome [RDS*, n* (%)]	19 (63%)	10 (67%)
Bronchopulmonary dysplasia [BPD, *n* (%)]	4 (13%)	2 (13%)
Regulatory problems at 3 months [*n* (%)]	13 (46%)^a^	6 (40%)
Regulatory problems at 6 months [*n* (%)]	7 (27%)^a^	6 (40%)
Regulatory problem at 12 months [*n* (%)]	7 (27%)^a^	7 (47%)
Regulatory problem persistency [*M* (SD)]	–	1.20 (1.26)
Age at uWECS assessment [months, *M* (SD)]	26.63 (24.31)	34.60 (22.25)
uWECS score [*M* (SD)]	7.06 (2.09)	7.43 (1.98)
Emotionally connected [yes, *n* (%)]	9 (30%)	5 (33%)
uWECS language [*n* (%)]		
German	23 (77%)	12 (80%)
Turkish	2 (7%)	2 (13%)
Russian	2 (7%)	–
Kurdish	2 (7%)	–
Twi	1 (3%)	1 (7%)
uWECS adult partner [*n* (%)]		
Biological mother	23 (77%)	12 (80%)
Biological father	5 (17%)	2 (13%)
Foster mother	2 (7%)	1 (7%)
Mothers’ education [ISCED level, *M* (SD)]	5.17 (2.02)	5.13 (1.92)
Fathers’ education [ISCED level, *M* (SD)]	5.28 (1.71)	5.20 (1.66)

^a^ Please note that only a subsample was assessed.

### Aim 1

An international, multiprofessional team of raters in Germany and Finland was trained remotely by MW and AH with weekly 1.5-hour sessions between October 2022 and February 2023 to administer and rate the uWECS. The training started with coding instructions for US-based dyadic interaction videos in English or Spanish language (see Hane et al., this edition). From December 2022, videos with participants from Germany were used. The training group members independently scored 10 videos for blinded reliability testing in February 2022. Reliability of *α *>* *.90 on the continuous dimensions and of kappa = 1.0 on the binary uWECS score with the standard raters (MW, AH) was achieved irrespective of language mismatches (i.e., all raters scored two to six videos with languages they did not understand). Next, the German-Finnish rating team scored the 30 dyads in the current sample (*n *= 23 spoke German, *n *= 7 spoke Russian, Turkish, Kurdish, or Twi during the interaction) and reliabilities were reassessed on group-level. Again, by design, there were language mismatches such that the German raters each scored seven videos they did not understand, while the Finnish rater understood none of the languages used in any of the interactions. Despite this heterogeneity, intra-class correlation coefficients (*ICCs*) among the five main raters for the four uWECS dimensions ranged from .98–.99. Dyads had mean uWECS scores of 7.06 (SD* *= 2.09, range 4.10–9.95, 30% were emotionally connected).

### Aim 2

**Figure 1:**
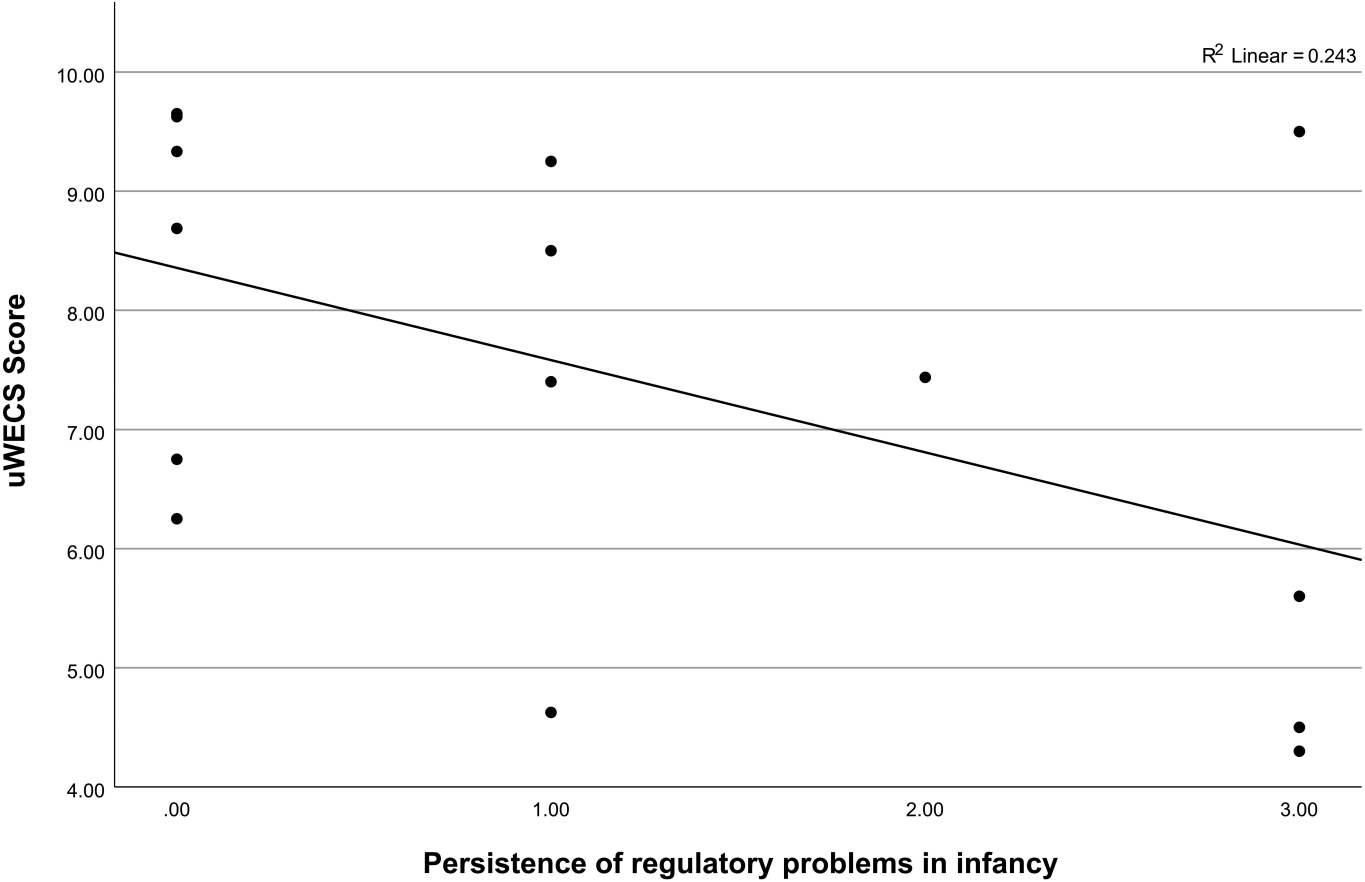
Association between the persistence of RPs during infancy and the uWECS score of mutual autonomic emotional connection (*n *= 15).

The 15 children who had been assessed longitudinally throughout infancy and childhood had mean RP scores of 1.20 (SD = 1.26). Dyads had mean uWECS scores of 7.43 (SD* *= 1.98, 33% were emotionally connected) at mean age 34.60 months (SD = 22.25, range 12–68 months). A multivariable linear regression analysis showed that the persistence of RPs in infancy (predictor of interest) was negatively associated with uWECS scores [*B* = -.82, SE = .29, *β*=-.53, *95%* CI* *= (−1.47, -.18), *p *= .017], after controlling for child sex and gestational age (see Table 2 and Figure 1).

**Table 2:** Associations between persistency of regulatory problems in infancy and uWECS scores (*n *= 15).

Dependent variable uWECS	*B*	SE	*β*	*p*	95% confidence interval for *B*
Regulatory problems	-.82	.29	-.53	.017	-1.47, -.18
Gestational age	.28	.09	.56	.011	.08, .48
Child sex	.95	.76	.23	.242	-.74, 2.63

## Discussion

This proof-of-concept study shows that the quality of mutual dyadic emotional connection among socio-culturally and linguistically heterogeneous samples can be reliably assessed with the brief 3-minute uWECS, despite a-piori planned language mismatches between raters and participants. In addition, confirming our hypothesis, pilot data showed that the persistence of RPs in infancy was negatively associated with the quality of emotional connection, after controlling for child sex and gestational age.

Calming cycle theory proposes that the ASR and autonomic emotional connection are fundamental building blocks of all mammalian behaviour (3). Accordingly, although acknowledging that each and every culture is shaped by unique contextual processes, it can be argued that a universal instrument such as the uWECS reliably captures the quality of mutual autonomic emotional connection across highly heterogeneous groups of dyads across diverse international contexts. Providing this proof-of-concept is critically important for evidence-based research and practice with globally growing cultural and linguistic diversity due to increased migration (38, 39). Indeed, although long neglected, the availability and implementation of equitable assessment tools that use a simple, universally accessible language has become paramount in the health sciences (40, 46). Moreover, it is well established that using their L1 in early dyadic interactions of parents with their children should be encouraged and facilitated (47–49). Therefore, following such recommendations that recognise the value and importance of families' linguistic and cultural heritage, mothers and fathers are always instructed to use their L1 during the uWECS interaction. Providing proof-of-concept that raters from very heterogeneous professional and language backgrounds can reliably code these 3-minute standardised interactions for emotional connection helps overcome the global lack of equitable, culture-fair screening tools (50) and associated language barriers, misunderstandings, and misdiagnoses.

Our results confirmed our hypothesis that more persistent RPs in infancy were prospectively associated with lower quality of mutual emotional connection between children and their mothers or fathers. Infant RPs represent both causes and consequences of broken emotional connection within a complex continuous feedback loop of co-regulatory changes that include foundational biological cycles such as signalling, sleep-wake, feeding, and breathing (3, 51, 52). Our pilot sample consisted of children born preterm with an average gestational age of 29.60 weeks, who had spent the first weeks of their lives in the neonatal intensive care unit (NICU). It is well documented that on top of the neurodevelopmental problems and medical complications triggered by preterm birth, the neonatal treatment itself causes pain and trauma that may affect the development of the stress response system (53, 54). At the same time, infants’ mothers also need to recover from the medical complications and trauma associated with a preterm birth, while both mothers and fathers face the stress of caring for a severely ill newborn. This may have long-term adverse consequences for the quality of mutual emotional connection and co-regulation (55, 56), and even result in severe consequences such as shaken baby syndrome (57). However, human nature has also equipped us with the ability to repair broken emotional connections (3, 7, 58), which deserves special attention among dyads at-risk for persistent dysregulation. The uWECS offers a valuable screening tool for early identification of suboptimal ASR and autonomic emotional connection, while its integrated treatment component, the Family Nurture Intervention, provides a seamless opportunity to help dyads reconnect and repair their natural ability to co-regulate (7, 59).

This study has several strengths but also limitations. We were able to provide proof-of-concept that a heterogeneous team of health care professionals can reach reliability in rating videorecordings of the brief uWECS interactions independent of language with only few remote sessions. The data from our clinical sample were collected prospectively and coded according to predefined protocols. A detailed documentation of participants' background characteristics allows comparison of their contextual experiences to other samples. However, overall our sample was very small, we did not assess mothers' and fathers' perceived daily stress levels or mental health which represent important confounders, and infant RPs were evaluated as part of an anamnestic approach during standard follow-up care. Replication of findings as part of large, socioeconomically and culturally diverse, prospective observational studies is warranted.

## Conclusion

This study provides proof-of-concept that the quality of dyadic autonomic emotional connection among linguistically diverse samples of infants and their parents can be reliably assessed with the uWECS, a brief screening that can be easily implemented in clinical practice. Pilot data suggests that persistent RPs during infancy are negatively associated with the quality of dyadic emotional connection. Replication of these findings in large and heterogeneous samples is needed. Future studies need to address how to facilitate successful emotion regulation for today's children and future generations.

## Data Availability

The datasets presented in this article are not readily available because there is no consent to the transfer of data to third parties for participants’ privacy right reasons. Requests to access the datasets should be directed to britta.huening@uk-essen.de.
