# Supplementary material for: Infant regulatory problems and the quality of dyadic emotional connection—a proof-of-concept study in a multilingual sample
Source: Front Child Adolesc Psychiatry. 2024 Jan 5;2:1304235. doi: 10.3389/frcha.2023.1304235 (PMC11731686; doi:10.3389/frcha.2023.1304235)
Supplement: Supplementary file 1 [file Datasheet1.pdf]

Infant regulatory problems and the quality of dyadic emotional connection – a proof-of-concept study in a multilingual sample

Jaekel, J., Dathe, A.-K., Brasseler, M., Bialas, J., Jokiranta-Olkoniemi, E., Reimann, M., Ludwig, R. J., Hane, A. A., Welch, M., & Huening, B.

Appendix 1. Parallel presentation of the full English and German uWECS positive and negative dimensional coding descriptions.

Appendix 2. The uWECS Scoring sheet in German

uWECS Positive Parallel English German

Translated by Nils Jäkel and Julia Jäkel

[“ATTRACTION”]

1. MOM AND CHILD BOTH WANT TO BE VERY CLOSE

– Child feels something very good because Mom’s body is touching his/her body. Mom feels the same.

Child very much wants to be close to Mom. Mom very much wants to be close to child.

– Mom often wants to see child's face. Mom feels something very good when she sees child’s face.
Child often wants to see Mom’s face. Child feels something very good when he/she sees Mom’s face.

– It is often like this for some time:
Mom looks at child’s eyes, at the same time child looks at Mom’s eyes. Mom feels something very good because of it. Child feels something very good because of it.

– Mom often touches child with her hands, Mom often touches child’s face with her face. When Mom does this, child feels something very good. At the same time Mom feels something very good.

– Child often touches Mom with his/her hands, child often touches Mom’s face with his/her face. When child does this, Mom feels something very good. At the same time, child feels something very good.

1. Mama und Kind wollen beide sehr nah sein

Kind fühlt etwas sehr Gutes weil Mamas Körper seinen Körper berührt. Mama fühlt dasselbe.

Kind will Mama sehr gerne nah sein. Mama will Kind sehr gerne nah sein.

Mama will oft Kinds Gesicht sehen. Mama fühlt etwas sehr Gutes wenn sie Kinds Gesicht sieht.
Kind will oft Mamas Gesicht sehen. Kind fühlt etwas sehr Gutes wenn es Mamas Gesicht sieht.

Es ist oft so für einige Zeit:
Mama sieht in Kinds Augen, zur selben Zeit sieht Kind in Mamas Augen. Mama fühlt deswegen etwas sehr Gutes. Kind fühlt deswegen etwas sehr Gutes.

Mama berührt Kind oft mit ihren Händen, Mama berührt Kinds Gesicht oft mit ihrem Gesicht. Wenn Mama das macht, fühlt Kind etwas sehr Gutes. Zur selben Zeit fühlt Mama etwas sehr Gutes.

Kind berührt Mama oft mit seinen Händen, Kind berührt Mamas Gesicht oft mit seinem Gesicht. Wenn Kind dies macht, fühlt Mama etwas sehr Gutes. Zur selben Zeit fühlt Kind etwas sehr Gutes.

[“COMMUNICATION”]

2. MOM AND CHILD BOTH WANT TO SAY SOMETHING TO THE OTHER. THEY BOTH WANT TO KNOW WHAT THE OTHER WANTS TO SAY

– Mom often says something to child. Mom can say it with words, she can say it with the face, she can say it with the eyes.
At the same time, Mom wants child to say something to her. When Mom thinks “Child wants to say something to me now,” Mom feels something very good.

– Child often says something to Mom. Child can say it with the face, he/she can say it with the eyes, he/she can say it with words.
At the same time, child wants Mom to say something to him/her. Child wants it very much.

– Often it is like this:
Mom says something good to child. Child feels something good because of it. After this, child says something good to Mom. Mom feels something good because of it.

– Often it is like this:
Mom says something to child, the next moment child says something to Mom. After this Mom says something more to child. When it is like this, child feels something very good, at the same time Mom feels something very good.

– Often it is like this:
When child says something about something, Mom wants to say more about the same thing.

– Sometimes child says with the face: “I see you now, I feel something good.” When child says something like this to Mom, child feels something very good, at the same time Mom feels something very good.

2. Mama und Kind wollen einander beide etwas sagen. Mama will wissen was Kind sagen will. Kind will wissen was Mama sagen will.

Mama sagt Kind oft etwas. Mama kann es mit Worten sagen, sie kann es mit Gesicht sagen, sie kann es mit Augen sagen.
Zur selben Zeit will Mama, dass Kind ihr etwas sagt. Wenn Mama denkt „Kind will mir jetzt etwas sagen“ fühlt Mama etwas sehr Gutes.

Kind sagt Mama oft etwas. Kind kann es mit Gesicht sagen, es kann es mit Augen sagen, es kann es mit Worten sagen.
Zur selben Zeit will Kind, dass Mama ihm etwas sagt. Kind will es sehr gerne.

Es ist oft so:
Mama sagt Kind etwas Gutes. Kind fühlt deswegen etwas Gutes. Danach sagt Kind Mama etwas Gutes. Mama fühlt deswegen etwas Gutes.

Es ist oft so:
Mama sagt Kind etwas, im nächsten Moment sagt Kind Mama etwas. Danach sagt Mama Kind etwas mehr. Wenn es so ist, fühlt Kind etwas sehr Gutes, zur selben Zeit fühlt Mama etwas sehr Gutes.

Es ist oft so:
Wenn Kind etwas über etwas sagt, will Mama mehr über dasselbe sagen.

Manchmal sagt Kind mit dem Gesicht: „Ich sehe dich jetzt, ich fühle etwas Gutes.“ Wenn Kind Mama so etwas sagt, fühlt es etwas sehr Gutes, zur selben Zeit fühlt Mama etwas sehr Gutes.

[“FACIAL COMMUNICATION”]

3. MOM AND CHILD LOOK AT THE OTHER'S FACE. THEY WANT TO KNOW WHAT THE OTHER FEELS

– Mom wants to know what child feels. Because of this, Mom often looks at child's face, she often looks at child's eyes.

– Child wants to know what Mom feels. Because of this, child often looks at Mom's face, child often looks at Mom's eyes.

– Often it is like this:
Mom says with the face: “I feel something good towards you”. Child says with the face: “I feel something good towards you”.

– Often it is like this:
Mom looks at child’s eyes for a long time. At the same time child looks at Mom’s eyes. When it is like this, Mom knows what child feels, at the same time child knows what Mom feels. Mom feels something very good because of it. Child feels something very good because of it.

3. Mama und Kind sehen einander ins Gesicht. Mama will wissen was Kind fühlt. Kind will wissen was Mama fühlt.

Mama will wissen was Kind fühlt. Deswegen sieht Mama oft Kinds Gesicht an, sie sieht oft in Kinds Augen.

Kind will wissen was Mama fühlt. Deswegen sieht Kind oft Mamas Gesicht an, es sieht oft in Mamas Augen.

Es ist oft so:
Mama sagt mit Gesicht: „Ich fühle etwas Gutes für dich“. Kind sagt mit Gesicht: „Ich fühle etwas Gutes für dich“.

Es ist oft so:
Mama sieht für eine lange Zeit in Kinds Augen. Zur selben Zeit sieht Kind in Mamas Augen. Wenn es so ist, weiß Mama was Kind fühlt, zur selben Zeit weiß Kind was Mama fühlt. Mama fühlt deswegen etwas sehr Gutes. Kind fühlt deswegen etwas sehr Gutes.

["SENSITIVITY/RECIPROCITY"]

4. MOM AND CHILD BOTH KNOW WHAT THE OTHER FEELS ALL THE TIME

– When child feels something at one moment, Mom often knows at the same moment what child feels. Mom's face often says: "I know what you feel now, I know what you want me to do, I want to do it."

When Mom feels something at one moment, child often knows at the same moment what Mom feels. Child's face often says: "I know what you feel now, I know what you want me to do, I want to do it."

– It is like this all the time:
If child feels something bad at one moment, Mom knows it. Mom doesn't want child to feel anything bad. Because of this, she says something good to child, she touches child. After this, child doesn't feel something bad anymore.

– It is like this all the time:
If Mom feels something bad at one moment, child knows it. Child doesn't want Mom to feel anything bad. Because of this, child says something good to Mom, he/she touches Mom. After this, Mom doesn't feel something bad anymore.

4. Mama weiß immer was Kind fühlt. Kind weiß immer was Mama fühlt.

Wenn Kind in einem Moment etwas fühlt, weiß Mama oft im selben Moment was Kind fühlt. Mamas Gesicht sagt oft: „Ich weiß was du jetzt fühlst, ich weiß was du willst was ich tun soll, ich will es tun."

Wenn Mama in einem Moment etwas fühlt, weiß Kind oft im selben Moment was Mama fühlt. Kinds Gesicht sagt oft: „Ich weiß was du jetzt fühlst, ich weiß was du willst was ich tun soll, ich will es tun."

Es ist immer so:
Wenn Kind in einem Moment etwas Schlechtes fühlt, weiß Mama es. Mama will nicht, dass Kind etwas Schlechtes fühlt. Deshalb sagt sie etwas Gutes zu Kind, sie berührt Kind. Danach fühlt Kind nichts Schlechtes mehr.

Es ist immer so:
Wenn Mama in einem Moment etwas Schlechtes fühlt, weiß Kind es. Kind will nicht, dass Mama etwas Schlechtes fühlt. Deshalb sagt es etwas Gutes zu Mama, es berührt Mama. Danach fühlt Mama nichts Schlechtes mehr.

["EMOTIONAL CONNECTION"]

5. MOM AND CHILD BOTH FEEL SOMETHING GOOD BECAUSE THEY ARE WITH THE OTHER

– Mom feels something very good because she is with child. When Mom feels something good, child knows it. Child feels something good because of it.

– Child feels something very good because he/she is with Mom. When child feels something good, Mom knows it. Mom feels something good because of it.

– It is like this:
When Mom feels something, child feels the same. When child feels something, Mom feels the same.

———

THIS IS VERY GOOD.
It is very good for Mom. It is very good for child.

5. Mama und Kind fühlen beide etwas sehr Gutes weil sie zusammen sind

Mama fühlt etwas sehr Gutes weil sie bei Kind ist. Wenn Mama etwas Gutes fühlt, weiß Kind das. Kind fühlt deswegen etwas Gutes.

Kind fühlt etwas sehr Gutes weil es bei Mama ist. Wenn Kind etwas Gutes fühlt, weiß Mama das. Mama fühlt deswegen etwas Gutes.

Es ist so:
Wenn Mama etwas fühlt, fühlt Kind dasselbe. Wenn Kind etwas fühlt, fühlt Mama dasselbe.

_ _ _

Dies ist sehr gut.
Es ist sehr gut für Mama. Es ist sehr gut für Kind.

uWECS Negative Parallel English German

Translated into German by Nils Jäkel and Julia Jäkel

[LACK OF "ATTRACTION"]

1. MOM AND CHILD DON'T WANT TO BE CLOSE

– Mom doesn't want to be very close to child. Mom doesn't feel something good when child's body is touching his/her body.
Child doesn't want to be very close to Mom. Child doesn't feel something good when Mom's body is touching his/her body.

– Mom doesn't often touch child with her hands. She doesn't touch child's face with her face.

– If Mom touches child, child doesn't want it. Child says "No" to Mom. Child can say it with words, child can say it with the face, child can say it with the body.
If child touches Mom, Mom doesn't want it. Mom says "No" to child. Mom can say it with words, she can say it with the face, she can say it with the body.

– Mom doesn't often look at child's face, Mom doesn't often look at child's eyes.
Child doesn't often look at Mom's face, child doesn't often look at Mom's eyes.

– When Mom doesn't look at child's face for a long time, child doesn't do anything. When child doesn't look at Mom's face for a long time, Mom doesn't do anything.

1. Mama und Kind wollen nicht nah sein

Mama will Kind nicht sehr nah sein. Mama fühlt nichts Gutes wenn Kinds Körper ihren Körper berührt.
Kind will Mama nicht sehr nah sein. Kind fühlt nichts Gutes wenn Mamas Körper seinen Körper berührt.

Mama berührt Kind nicht oft mit ihren Händen. Sie berührt Kinds Gesicht nicht mit ihrem Gesicht.

Wenn Mama Kind berührt will Kind es nicht. Kind sagt Mama „Nein". Kind kann es mit Worten sagen, Kind kann es mit Gesicht sagen, Kind kann es mit Körper sagen.
Wenn Kind Mama berührt will Mama es nicht. Mama sagt Kind „Nein". Mama kann es mit Worten sagen, sie kann es mit Gesicht sagen, sie kann es mit Körper sagen.

Mama sieht Kinds Gesicht nicht oft an, Mama sieht nicht oft in Kinds Augen.
Kind sieht Mamas Gesicht nicht oft an, Kind sieht nicht oft in Mamas Augen.

Wenn Mama eine lange Zeit Kinds Gesicht nicht ansieht, tut Kind nichts. Wenn Kind eine lange Zeit nicht Mamas Gesicht ansieht, tut Mama nichts.

[LACK OF “COMMUNICATION”]

2. MOM AND CHILD DON’T SAY MUCH TO THE OTHER

– Mom doesn’t often say something to child. Mom doesn’t say anything with the face, she doesn’t say anything with the eyes.
Child doesn't often say something to Mom. Child doesn't say anything with the face, child doesn't say anything with the eyes.

– When Mom says something to child, child doesn’t want it. Child says “No” to Mom. Child can say it with words, child can say it with the face.
When it is like this, child feels something bad. At the same time, Mom feels something bad.

– When Mom doesn’t say anything to child for a long time, child doesn't do anything. When child doesn’t say anything to Mom for a long time, Mom doesn't do anything.

2. Mama und Kind sagen einander nicht viel

Mama sagt Kind nicht oft etwas. Mama sagt nichts mit Gesicht, sie sagt nichts mit Augen.
Kind sagt Mama nicht oft etwas. Kind sagt nichts mit Gesicht, Kind sagt nichts mit Augen.

Wenn Mama Kind etwas sagt, will Kind es nicht. Kind sagt Mama „Nein“. Kind kann es mit Worten sagen, Kind kann es mit Gesicht sagen.
Wenn es so ist, fühlt Kind etwas Schlechtes. Zur selben Zeit fühlt Mama etwas Schlechtes.

- Wenn Mama Kind eine lange Zeit nichts sagt, tut Kind nichts. Wenn Kind Mama eine lange Zeit nichts sagt, tut Mama nichts.

[LACK OF “FACIAL COMMUNICATION”]

3. MOM AND CHILD DON’T LOOK AT THE OTHER’S FACE

– Mom doesn’t often look at child's face.
Child doesn't often look at Mom's face.

– It is not like this:
Mom wants to see child’s face, Mom wants to see child’s eyes.
Child wants to see Mom’s face, child wants to see Mom’s eyes.

– Child doesn’t want Mom to look at his/her face. When Mom looks at child’s face, sometimes child turns away. Sometimes child says with the face “I feel something bad towards you”.

Mom doesn’t want child to look at her face. When child looks at Mom’s face, sometimes Mom turns away. Sometimes Mom says with the face: “I feel something bad towards you”.

– It is never like this:
Child looks at Mom’s face for some time, at the same time Mom looks at child’s face.

3. Mama und Kind sehen einander nicht an

Mama sieht Kinds Gesicht nicht oft an.
Kind sieht Mamas Gesicht nicht oft an.

Es ist nicht so:
Mama will Kinds Gesicht sehen, Mama will Kinds Augen sehen.
Kind will Mamas Gesicht sehen, Kind will Mamas Augen sehen.

Kind will nicht, dass Mama sein Gesicht ansieht. Wenn Mama Kinds Gesicht ansieht, dreht Kind sich manchmal weg. Manchmal sagt Kind mit Gesicht „Ich fühle etwas Schlechtes für dich“.

Mama will nicht, dass Kind ihr Gesicht ansieht. Wenn Kind Mamas Gesicht ansieht, dreht Mama sich manchmal weg. Manchmal sagt Mama mit Gesicht „Ich fühle etwas Schlechtes für dich“.

Es ist nie so:
Kind sieht für einige Zeit Mamas Gesicht an, zur selben Zeit sieht Mama Kinds Gesicht an.

[LACK OF “SENSITIVITY/RECIPROCITY”]

4. MOM AND CHILD DON’T KNOW WHAT THE OTHER FEELS

– When child feels something, Mom doesn't know it. Mom's face doesn't say: “I know what you feel now.”
When Mom feels something, child doesn’t know it. Child’s face doesn’t say: “I know what you feel now.”

– It is like this all the time:
If child feels something bad at one moment, Mom doesn’t know it. When child feels something bad, Mom doesn’t say something good to child. Mom doesn’t touch child, Mom doesn’t do anything else.

– It is like this all the time:
If Mom feels something bad at one moment, child doesn’t know it. When Mom feels something bad, child doesn’t say something good to Mom. Child doesn’t touch Mom, child doesn’t do anything else.

– Sometimes it is like this:
Child wants to know what Mom feels. Because of this, child looks at Mom's face for a very short time. Child doesn’t know what Mom feels. After some time, child looks at Mom’s face again for a very short time. It happens like this many times.

4. Mama weiß nicht was Kind fühlt. Kind weiß nicht was Mama fühlt.

Wenn Kind etwas fühlt, weiß Mama es nicht. Mamas Gesicht sagt nicht: „Ich weiß was du jetzt fühlst.“
Wenn Mama etwas fühlt, weiß Kind es nicht. Kinds Gesicht sagt nicht: „Ich weiß was du jetzt fühlst.“

Es ist immer so:
Wenn Kind in einem Moment etwas Schlechtes fühlt, weiß Mama es nicht. Wenn Kind etwas Schlechtes fühlt, sagt Mama Kind nichts Gutes. Mama berührt Kind nicht, Mama tut nichts anderes.

Es ist immer so:
Wenn Mama in einem Moment etwas Schlechtes fühlt, weiß Kind es nicht. Wenn Mama etwas Schlechtes fühlt, sagt Kind Mama nichts Gutes. Kind berührt Mama nicht, Kind tut nichts anderes.

Es ist manchmal so:
Kind will wissen was Mama fühlt. Deswegen sieht Kind Mamas Gesicht für eine sehr kurze Zeit an. Kind weiß nicht was Mama fühlt. Nach einiger Zeit sieht Kind Mamas Gesicht wieder für eine sehr kurze Zeit an. So passiert es viele Male.

[LACK OF "EMOTIONAL CONNECTION"]

5. MOM AND CHILD DON'T FEEL SOMETHING GOOD BECAUSE THEY ARE WITH THE OTHER

– It is not like this:
Mom feels something good because she is with child. Child feels something good because he/she is with Mom.

– It is not like this:
When Mom feels something, child feels the same. When child feels something, Mom feels the same.

———

THIS IS NOT GOOD.
This is not good for Mom. This is not good for child.

It will be good if someone speaks to Mom about it, someone like a doctor.

5. Mama und Kind fühlen beide nichts Gutes weil sie zusammen sind

Es ist nicht so:
Mama fühlt etwas Gutes weil sie bei Kind ist. Kind fühlt etwas Gutes weil es bei Mama ist.

Es ist nicht so:
Wenn Mama etwas fühlt, fühlt Kind dasselbe. Wenn Kind etwas fühlt, fühlt Mama dasselbe.

– – –

Dies ist nicht gut.
Dies ist nicht gut für Mama. Dies ist nicht gut für Kind.

Es wird gut sein wenn jemand mit Mama darüber spricht, jemand wie ein Arzt.

V.1

uWECS SCORE SHEET

GERMAN - DEUTSCH

Universal Welch Emotional Connection Screen - clear explicit language

Kind auf dem Schoß des Elternteils, sie sehen einander an, 3 Minuten lang.
Keine Gegenstände, Spielzeuge, Lebensmittel.

1

Mama und Kind wollen beide sehr nah sein

1	1.25	1.5	1.75	2	2.25	2.5	2.75	3

2

Mama und Kind wollen einander beide etwas sagen.
Mama will wissen was Kind sagen will. Kind will wissen was Mama sagen will.

1	1.25	1.5	1.75	2	2.25	2.5	2.75	3

3

Mama und Kind sehen einander ins Gesicht.
Mama will wissen was Kind fühlt. Kind will wissen was Mama fühlt.

1	1.25	1.5	1.75	2	2.25	2.5	2.75	3

4

Mama weiß immer was Kind fühlt. Kind weiß immer was Mama fühlt.

1	1.25	1.5	1.75	2	2.25	2.5	2.75	3

Fühlen Mama und Kind beide etwas sehr Gutes weil sie zusammen sind?

☐ **Nein** ☐ **Ja**
